# An American Text Book of the Diseases of Children

**Published:** 1899-06

**Authors:** 


### Books and Magazines.

An American Text Book of the Diseases of Children, including chapters on essential surgical subjects; orthopædics; diseases of the Eye, Ear, Nose and Throat; diseases of the Skin, and on the diet, hygiene and general management of children. By American Teachers. Edited by Louis Starr, M. D., Consulting Pædiatrist to the Maternity Hospital, etc.; and Thompson S. Wescott, M. D., Instructor in Diseases of Children, University of Pennsylvania, etc. Second edition; revised. Pages, 1240; cloth, $7.00; sheep or half morocco, $8.00. By subscription only. Published by W. B. Saunders, Philadelphia, 1898.

This great work is justly regarded with pride by the entire profession in this country, and is a monument to American medical learning as well as to the enterprise of one of our leading medical publishing houses. The revised edition is a great improvement, is a distinct improvement on the first. New articles have been added, and many of the old have been entirely rewritten, and all have been emended in order to bring such subject thoroughly up to date.

The new articles include "Modified Milk and Percentage Milk Mixtures;" Lithæmia, and an entire section on Orthopædics. Those rewritten are "Typhoid Fever," "Rubeola," "Chicken Pox," "Tuberculous Meningitis," "Hydrocephalus" and "Scurvy." The volume has been increased by fifty pages of new matter. It has been the intention of the publisher, in bringing out this series of American text books, to furnish in compact and portable volumes, working guides to the practitioner, and not voluminous reference works, and yet include in them all necessary knowledge of the subject. That he has succeeded in his effort is attested by the great popularity of the series, which includes volumes on Eye, Ear, Throat and Nose; Genito-Urinary and Skin Diseases, Gynecology, Applied Therapeutics, Legal Medicine and Toxicology; Nursing; Obstetrics; Pathology; Physiology; Theory and Practice, Surgery, and the volume under review. These volumes furnish one of the most complete and valuable medical libraries ever published, and the enterprising house has conferred a benefit upon the profession that should merit their thanks and substantial support. Too much cannot be said in praise of the present volume on diseases of children. Its contents are too numerous and varied to permit separate mention in the space available for this notice. Suffice to say that the scope is ample, the treatment of each topic has been entrusted to that member of the profession in any part of this country who was best fitted to discuss it, and the whole has been revised and adapted by one of the ablest pædiatrists the country can boast of. J.

